# Resolvin D1 shows osseous-protection *via* RANK reduction on monocytes during orthodontic tooth movement

**DOI:** 10.3389/fimmu.2022.928132

**Published:** 2022-10-07

**Authors:** Yehuda Klein, Offir Levin-Talmor, Jaime Garber Berkstein, Sharon Wald, Yaron Meirow, Avi Maimon, Avi Leibovich, Yechezkel Barenholz, David Polak, Stella Chaushu

**Affiliations:** ^1^ Department of Orthodontics, Faculty of Dental Medicine, Hadassah Medical Center, Hebrew University of Jerusalem, Jerusalem, Israel; ^2^ Department of Biochemistry, Israel–Canada Medical Research Institute, Faculty of Medicine, Hebrew University of Jerusalem, Jerusalem, Israel; ^3^ The Institute of Dental Sciences, Hebrew University of Jerusalem, Jerusalem, Israel; ^4^ Lautenberg Center for General and Tumor Immunology, Israel–Canada Medical Research Institute, Faculty of Medicine, Hebrew University of Jerusalem, Jerusalem, Israel; ^5^ Faculty of Dental Medicine, Hebrew University of Jerusalem, Jerusalem, Israel; ^6^ Department of Periodontics, Hadassah Medical Center, Jerusalem, Israel

**Keywords:** Resolvin D1 in orthodontic tooth movement, orthodontic tooth movement, immunomodulation, bone remodeling, osteoclastogenesis, Resolvin D1

## Abstract

The study aimed to investigate the role of RvD1 in acute and prolonged sterile inflammation and bone remodeling. A mouse model of sterile inflammation that involves bone resorption was used to examine endogenous RvD1 kinetics during inflammation. Application of exogenous RvD1 significantly inhibited bone remodeling *via* osteoclast reduction, alongside an anti-inflammatory secretome shift, increased macrophages recruitment and reduction of T-cytotoxic cells. *In vitro* and *in vivo*, RvD1 led to significant reduction in RANK expression which reduce osteoclastogenesis in a dose-dependent manner. Taken together, the data shows a dual role for RvD1, as a potent immunoresolvent agent alongside an osteoresolvent role, showing a potential therapeutic agent in bone resorption associated inflammatory conditions.

## Introduction

Orthodontic tooth movement (OTM) is produced by a mechanical force which triggers an acute inflammatory process driven by immune cells and mediators. This ignites alveolar bone remodeling (BR) mainly *via* the receptor activator of nuclear factor kappa-Β ligand (RANKL)/RANK/osteoprotegerin (OPG) axis, enabling tooth displacement (1). To depict the extensive role of the immune system in OTM the term “Immunorthodontics” has been recently introduced (2).

Acute inflammation is the defense and vital immune system’s response to injury, which aims to minimize damage, promote resolution of inflammation and restoration of tissue homeostasis. Under healthy conditions, resolution occurs without any external intervention, *via* an active process, orchestrated by specialized proresolving mediators (SPMs) of inflammation, including resolvins, lipoxins, protectins, and maresins (3). Failure of resolution leads to uncontrolled inflammation which contributes to a variety of chronic inflammatory diseases (4, 5).

OTM has been classically divided into 4 phases: initial, arrest, acceleration, and linear. In the initial phase, the mechanical force triggers an acute inflammatory sterile reaction which ignites bone resorption and tooth movement within the alveolar socket. The next lag (arrest) phase occurs due to formation of a local necrotic area in the periodontal ligament (PDL) and blockage of the surrounding alveolar bone. During this period, the acute inflammation underlying the initial phase usually dampens despite continuous force delivery. Bone resorption continues, but some areas of bone formation start to appear. This coupling mechanism between resorption and formation is crucial as it ensures maintaining the bone volume after the previous phase in which resorption predominated. Recently, the existence of an active resolution process underlying the lag phase has been proposed. With continuous optimal force delivery, the resolution turns into a low-grade chronic inflammation in the next acceleration and linear phases, in which bone remodeling and tooth movement proceed at a constantly increased and then at a steady state rate (6).

Based on this assumption, we hypothesized that by immunomodulating the resolution process, BR and OTM rate might be successfully controlled.

Resolvins are a group of endogeneous lipid mediators produced during the resolution phase of acute inflammation from Eicosapentaenoic acid and Docosahexaenoic acid with two chemically unique structural forms, the E-series and D-series. They possess dual anti-inflammatory and pro-resolving activities which help preventing progression of an acute inflammatory response into chronic inflammation (7).

Resolvin D1 (RvD1) was shown to reduce polymorphonuclear leukocyte (PMN) infiltration in acute inflammation, suppress excessive pro inflammatory and encourage anti-inflammatory mediator production, promote clearance of apoptotic PMNs and regulate macrophage function (3, 7–10). RvD1 was also shown to reduce cytokine induced production of PGE2 and upregulate LXA4 production by PDL cells and monocytes, enhance PDL fibroblast proliferation and wound closure, thereby promoting PDL regeneration (11).

In the context of osteoimmunology, most studies focused on the inhibitory effect of RvE1 on inflammatory bone resorption, which has been attributed to a decreased production of RANKL, as well as to downregulation of osteoclast differentiation (12–17).

In contrast, only few studies investigated the effect of RvD1 on bone resorption. Vasconcelos et al. proved that RvD1 had a positive role in bone repair of rat femoral defects (18). Similarly, Benabdoun et al. showed that RvD1 inhibited bone resorption triggered by autoimmune inflammation in arthritic mice (19).

The aims of the present study were to study the effect of RvD1 in OTM induced BR, unraveling its biological mechanisms of action. The findings will provide an insight into the biology underlying the OTM resolution phase, paving the way for development of novel immunomodulatory therapies to control its rate, avoiding undesirable movement of anchorage teeth and posttreatment relapse.

## Materials and methods

### Animals and ethics

The study was approved by the IACUC of the Hebrew University (MD-18-15426-4) and conforms to the ARRIVE guidelines. C57BL mice (male 8-9-week-old, 23 ± 2 gr) were purchased from Harlan (Jerusalem, Israel). Only male mice were used to avoid the effect of the sex cycle and hormonal changes (20). The animals were housed in the Specific Pathogen-Free Facility of the Hebrew University, kept at 25°C with a 12/24h light/dark cycle and fed a granular diet. Body weight and health were monitored every other day.

### The OTM mouse model of sterile inflammation induced bone remodeling

To investigate the role of RvD1 in sterile inflammation and bone remodeling, an OTM mouse model was chosen (2, 21). Briefly, mice (*n* = 6-8/time point) were anesthetized with intraperitoneal (IP) injection of ketamine (200 mg/kg) and xylazine (10 mg/kg) at a 9:1 ratio, respectively. In addition, a dose of 10 µl of I0.4% lidocaine was injected for local anesthesia (22). Following anesthesia, a 3mm NiTi closed coil spring (10g; TOMY International, Tokyo) was inserted between the upper incisors and the upper left first molars (ULM1), generating a constant force (calibrated by gauge, data not published) and a mesial movement of the ULM1. In the experiments including exogenous RvD1 administration, all injections were administered subperiosteally adjacent to the mesial surface of the ULM1, using a microliter syringe, 26-gauge needle (Hamilton Company) as previously described (23), with minor modifications. Depending on the experiment, the control groups included animals with inactive springs or animals with active springs and saline administration.

### Evaluation of the RvD1 endogenous levels in the acute phase of sterile inflammation

In order to examine whether RvD1 plays a role in sterile inflammation, the treatment site was harvested at 6 and 24 hours post force application (OTM model) and compared with the control group of mice with inactivated springs (n=6/group). These time points were selected based on previous articles which showed that the acute inflammatory process and its subsequent resolution occur in the first 24 hours post force application (2). Following euthanization, the gingival mucosa was removed and the left hemi-maxillary bone specimens were collected in 300µl of PBS contained beads and homogenized (3 cycles, 5 minutes each) with a Standard Homogenizer (Bullet Blender^®^). Following centrifugation (10000g, 10 minutes, 4°C), the lysates were used to detect endogenous levels of RvD1, using RvD1 ELISA Kit (Cayman Chemical^®^), according to the manufacturer’s instructions. The assay had a range from 3.3-2,000 pg/ml and a sensitivity (80% B/B0) of approximately 15 pg/ml.

### Evaluation of the effect of exogeneous RvD1 on OTM associated bone remodeling

#### a. Radiographic analysis

The amount of OTM was measured at 3- and 14-days post force application. After euthanization, the gingival mucosa and the springs were removed; maxillary bone specimens were collected, fixed in 4% paraformaldehyde overnight and dehydrated with 70% ethanol. Samples were scanned by the Micro CT scanner (µCT40^®^, SCANCO, Switzerland) at 70 kVp, 114µA intensity, and 1,000 projection at a 200-ms integration time, as previously described (24–26). Two- and three-dimensional images were constructed. The amount of OTM was measured as the distance between the height of contours of the first and the second left maxillary molars, as previously described (2).

#### b. Histomorphometry staining

The maxillae were fixed overnight at 4°C in 4% paraformaldehyde/PBS solution, washed for 1 week in 10% EDTA, cryopreserved in 30% sucrose (overnight at 4°C), embedded in Optimal Cutting Temperature (OCT) and finally cryo-sectioned into 10-μm-thick sections (25). The sections were conventionally stained for tartrate-resistant acid phosphatase (TRAP) staining (Sigma Aldrich kit^®^). Slides were analyzed under light microscope Olympus BX45 (Olympus, Central Valley, PA) at x4, x10 and x20 magnification. Osteoclasts were identified as TRAP-positive, multinucleated cells located on the bone surface. The area around the mesio-buccal root of the maxillary first molar was divided into mesial and distal sides and the number of TRAP-positive cells *per* 1000 µm^2^ of PDL and adjacent alveolar bone (excluding the marrow cavities and blood vessels), was counted.Proteome Profiler Array and Bicinchoninic acid (BCA) assay: To characterize site secretome following RvD1 administration in the sterile inflammation model, we used the Proteome Profiler™ Mouse XL cytokine Array (R&D systems^®^). In brief, following 24h of inflammation, mice were sacrificed, and gingival mucosa was removed. The left hemi- maxillary bone specimens were homogenized with Protease and Phosphatase inhibitor cocktail (Sigma) and the total protein concentration was first measured using BCA assay (Thermo Scientific^©^). Following protein normalization, a pool for each group was prepared (OTM w/o RvD1 injections, n=5-6 mice/group) and the Proteome Profiler™ array was used according to manufacturer instructions. Analysis was carried out with Image Lab™ software. Since this array is semi-quantitative, an ELISA for IL1-ra and CCL6 was performed to validate the results.

#### c. Enzyme-linked immunosorbent assay

To validate Proteome Profiler Array, three selected cytokines including IL-1ra, IL-6 and CCL6/10 (R&D systems^®^) (singled out according to the Profiler Array results) were further validated with ELISA. Briefly, after BCA assay normalization, samples were analyzed as individual values for each mouse in each group separately. The absorbance was measured at 540nm wavelength using micro plate reader (Bio-Tek Instruments, Winooski, VT, USA) according to the manufacturer instructions.

#### d. Immunotyping of PDL

Immunotyping of the PDL tissue by flow cytometry (FACS) analysis was performed as previously described (22). Prior to OTM and following anesthesia, 21 mice (n=7/group) were divided into 3 groups: (a) experimental group which received a single sub-gingival injection of 10μl of 0.1 mg/ml RvD1 (b) control (sham) group which received a single sub-gingival injection of 10μl saline (c) control group with inactivated springs and no subgingival injections. Mice were sacrificed after 24 hours and the ULM1 teeth were gently extracted and incubated in working solution containing PBS (x1), 2% Fetal Calf Serum (Sigma Aldrich), Collagenase 2 (1μg/ml; Sigma Aldrich) and DNase (1μg/ml; Sigma Aldrich) for 25 minutes at 37°C on a shaker. Following incubation, EDTA 0.5M was added to working solution. The solutions were collected, filtered and centrifuged (1400 rpm; 8 minutes; 4°C). Aliquots of the samples were divided into experimental groups and stained with fluorochrome-conjugated monoclonal antibodies: CD45, CD3, Ly-6G, CD8, F4/80, CD64, CD265 (BioLegend^®^) for the detection of leukocytes, T cells, neutrophils, T cytotoxic cells, macrophages, monocytes and Receptor Activator of Nuclear Factor κB (RANK); respectively. Following incubation, samples were analyzed using the BD LSR Fortessa™ cell analyzer.

#### e. Immunofluorescence staining

To verify the FACS results following 1 day of OTM and RvD1 administration, IF staining was performed. Briefly, the cryo-sectioned slides (detailed above) were washed in PBS, embedded in warm antigen‐retrieval citrate buffer (Abcam, Cambridge, MA) for 20 min and washed three times with cold PBS. Next the slides were washed with Tris buffer (TBS) containing 0.025% Triton X‐100 and blocked in 1% bovine serum albumin (BSA) in TBS for 1 hour at room temperature. Then the slides were incubated overnight at 4°C with primary antibodies: Monocyte (CD64), Macrophage (F4/80) and RANK (CD265) in PBS containing 1% BSA (all purchased from Abcam). Samples were incubated with a secondary antibody conjugated to a fluorophore –Donkey Anti-Rat IgG (purchased from Abcam) in PBS with 1% BSA for 1 hour at room temperature, washed three times with TBS and counter‐stained with 4′, 6‐diamidino‐2‐phenylindole (DAPI). The samples were sealed with Gel Mount Aqueous (Sigma‐Aldrich). Negative staining controls included slides from which the primary antibody was omitted. The samples were analyzed under a fluorescence microscope (Nikon TL, Tochigi, Japan).

### Repeated exogenous RvD1 delivery procedure (prolonged inflammation)

Following anesthesia, the experimental OTM group received subgingival injections of 10μl of either 0.1 mg/ml RvD1 or saline, every other day, for a period of 14 days. These doses were calculated according to Lee et al, with adaptation to mice (17). The control group in this experiment included mice with inactive springs and no subgingival injections.

### *In vitro* experiments

#### Cell cultures

Murine macrophage RAW 264.7 cells (ATCC, Manassas, VA, USA) were cultured in Corning^®^ 75cm^2^ Cell Culture Flask for 3 days in α- Modified Eagle Medium (α-MEM, Biological Industries^©^) supplemented with 10% Fetal Bovine Serum (FBS) and 1% antibiotics (Penicillin-Streptomycin). Flasks were incubated in 37°C humidified incubator gassed with 5% CO_2_. Medium was changed every other day until 70% cell confluence was reached. Cells were mechanically scraped, counted and seeded in 96-well tissue culture plates at density of 5x10^5^ cells per well in the presence of soluble Receptor Activation of Nuclear factor-ĸB Ligand (RANKL) at a final concentration of 25ng/ml.

#### RAW 264.7 cells stimulation with RvD1

In order to examine the direct effect of RvD1 on osteoclast formation, RAW 264.7 cells were stimulated with different doses (2, 20 and 200 ng/ml) of RvD1 (Cayman Chemical^®^) throughout the 3 days incubation period with the RANKL (27). Cells treated with RANKL and without RvD1 served as a positive control group for osteoclast formation.

#### Tartrate-resistant acid phosphatase staining for cell culture

At 3 days of incubation with RANKL, cells were fixed with 4% Paraformaldehyde solution for 10 minutes and stained for Tartrate-Resistant Acid Phosphatase (TRAP) staining kit (Sigma Aldrich^®^) according to manufacturer instructions. Digital images of TRAP positive cells were done using a binocular microscope (Zeiss Axiovert 200). The TRAP positive multinucleated cells that contain three or more nuclei were counted as osteoclasts. Data is displayed as the average number of multinucleate TRAP positive cells per well.

#### Viability assay

To evaluate the impact of RvD1 on cell viability, proliferation, and cytotoxicity, following 3 days of RANKL and RvD1 incubation, a commercial XTT assay kit (Biological Industries^©^) was used according to the manufacturer’s instructions. Results are presented as optical density value after subtraction of blank reading.

#### siRNA transfection of cells

In order to confirm that the RvD1 effect is solely mediated by RANK, we used the siRNA assay, which specifically reduce RANK pathway. Cells were transfected with 2 siRNA sequences: one was targeted to RANK (Dharmacon siGENOME Mouse pure individual siRNAs (sense 5′-GCGCAGACUUCACUCCAUAUU-3′, antisense 5′-UAUGGAGUGAAGUCUGCGCUU-3′), previously validated on RAW 264.7 cells and the other was a non-targeting control siRNA (sense: 5′-UAGCGACUAAACACAU CAAUU-3′, antisense: 5′- UUAUCGCUGAUUUGUGUAGUU-3′) both used with a cationic lipid cell transfection reagent (DharmaFECT 4) (28).

RAW cells were seeded at density of 1×10^4^ cells per well in 96-well plates in α-MEM medium at 37°C with 5% CO_2_ overnight. Transfection reagent DF4 and siRNA were prepared according to manufacturer’s instructions. Final dosing concentrations of all siRNAs provided to each well were 0.5 µM in a total volume of 0.2 µL DF4. Transfection with siRNA/DF4 complexes was carried out in complete media. Subsequently, siRNA transfection was immediately performed. Cells uptake of siRNA complexes was performed by incubating cells with siRNA complexes in complete media at 37°C with 5% CO_2_. In order to generate osteoclasts, cells were transfected by siRNA complexes in complete media with a final concentration of 25 ng/ml of RANKL. Non-specific knock-down of DF4 served as a control and was assessed by using non- targeting siRNA dosed under identical conditions. Following 24 hours of incubation, cells were fixed with 4% Paraformaldehyde solution, stained with CD265 antibody and DAPI. RANK expression was measured with a florescent plate reader (Tecan^©^ M200 Plate Reader). Digital images were also taken using a fluorescence microscope (Nikon TL, Tochigi, Japan).

An additional experiment was carried out to compare osteoclastogenesis; siRNA transfected cells were incubated with RANKL. Controls included sham and RvD1 treated cells. After 3 days of incubation with RANKL, cells were fixed with 4% Paraformaldehyde solution for 10 minutes and stained using TRAP staining kit (Sigma Aldrich^®^) according to manufacturer instructions.

#### Immunofluorescence staining for receptor activator of nuclear factor κ

To measure the possible direct effect of RvD1 on pre-osteoclast cells, we chose to evaluate the RANK expression on RAW cells w/o RvD1treatment by using a PE- anti mouse CD265 (RANK) IF Abs. In brief, following 1 and 3 days of RANKL incubation, cells were fixed with 4% PFA for 10 minutes, stained with 1% CD265 antibody for 45 minutes, and counterstained with 1% DAPI for 5 minutes. RANK expression was measured with a florescent plate reader (Tecan^©^ M200 Plate Reader). To visualize, digital images were also taken using a fluorescence microscope (Nikon TL, Tochigi, Japan).

#### Statistical analysis

All analyzes were done with SPSS Version 10 software (SPSS, Inc., NY, NY). Power calculations showed that a minimum of 6 animals were needed for each group (power *>* 0.8). The data were analyzed according to Student’s one tailed unpaired t-test (unequal variances assumed). Significance levels: *= *p<*0.05, **= *p<*0.01, ***= *p<*0.001, ****= *p<*0.0001. The data are presented as mean ± SD.

## Results

### RvD1 plays an active role in OTM

**Figure 1:**
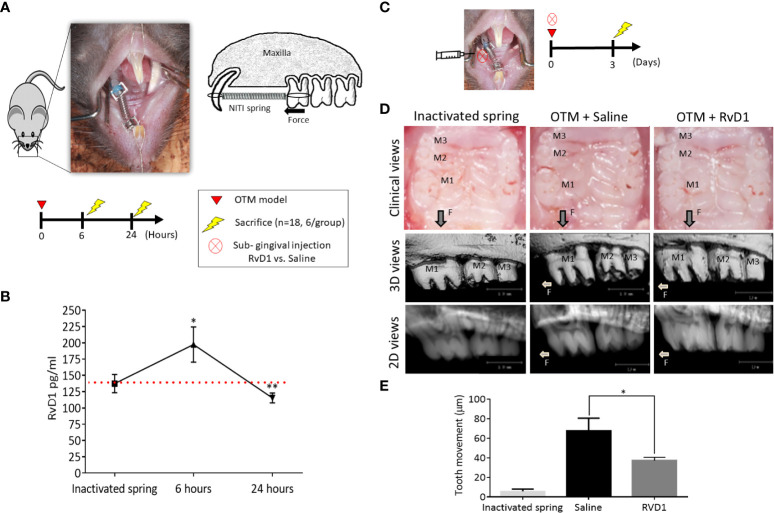
RvD1 Plays an Active Role in OTM **(A)** Schematic time course of endogenous RvD1 levels, after 6 and 24 hours of OTM compared to inactivated spring control group (n=6/group). **(B)** Endogenous levels of RvD1 during the initial phase of OTM. Red line represents the endogenous baseline level of RvD1 as measured in the inactivated spring group. **(C)** Schematic time course of OTM + single subgingival injection of 10μl of either 0.1 mg/ml RvD1 vs. Saline and inactivated spring without act (n=6/group), after 3 days. **(D)** OTM post force application + single subgingival injection of RvD1 vs. Saline and inactivated spring, after 3 days. Clinical views of the treated maxillae (top); 3D images taken by µCT (middle); 2D images taken by µCT (bottom). **(E)** OTM analysis (*p<0.05).

To determine whether RvD1 has an active role during initial phases of OTM, we examined the endogenous levels of RvD1 in the OTM model, 6- and 24-hours post force application. Mice with inactivated springs served as controls (**Figure 1A**). An increase in endogenous RvD1 levels was observed at 6 hours in comparison to the inactivated spring (197.4 ± 27.1 pg/ml versus 137. 5 ± 13. 9 pg/ml, respectively; P<0.05). After 24 hours, RvD1 significantly decreased to levels below the baseline of the inactivated spring group (115.4 ± 7.5 pg/ml; P<0.01) (**Figure 1B**). These results indicate an active role for endogenous RvD1 in the acute phase of OTM-induced inflammation.

Next, we evaluated the *in vivo* effect of RvD1, by including exogenous sub-gingival injections. After 3 days of force application and a single RvD1 injection, clinical views showed a reduction in OTM in comparison to saline administration (**Figure 1C**). Radiographic measurements (**Figure 1D**) confirmed the clinical views (**Figure 1D**), with 37 ± 3.5 μm after single RvD1 injection versus 67.3 ± 13.2 μm in the saline injection control group (p<0.05) (**Figure 1E**).

### RvD1 affects the extracellular secretome composition in the acute OTM phase

**Figure 2:**
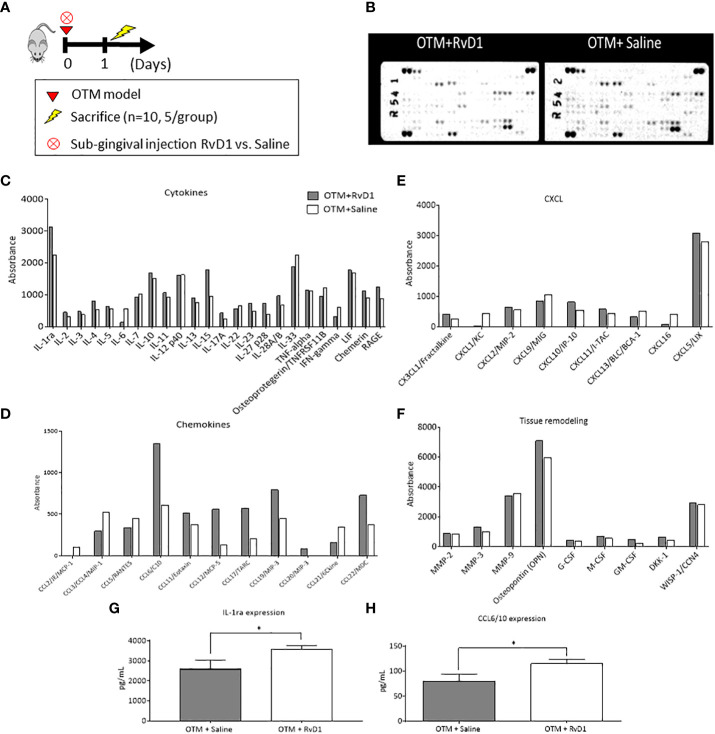
RvD1 Affects the Extracellular Secretome Composition in the Acute Phase of OTM Extracellular Secretome microarray performed on total lysates of OTM sites following RvD1 or Saline treatment after 1 day. Protein expression levels are presented as arbitrary units measured by densitometry. n = 5 mice/group. For the array experiment, they mice were pooled. ^∗^p < 0.01. **(A)** Experimental design. **(B)** Array images of the 2 membranes showing the positive signals seen on developed films; proteins are subcategorized for analysis: **(C)** Cytokines expression; **(D)** Chemokines expression; **(E)** CXCL expression; **(F)** Tissue remodeling factors expression. *All numbers were normalized/standardized according to the reference points. **(G)** IL-1ra protein levels (pg/mL) in mice treated with OTM+RvD1 vs. Saline for 1 day, obtained by ELISA (*p<0.05). **(H)** CCL6/10 protein levels (pg/mL) of mice treated with OTM+RvD1 vs. Saline for 1 day, obtained by ELISA (*p<0.05).

The acute inflammation in OTM is controlled by extracellular signaling molecules, such as cytokines, chemokines and growth factors which affect cellular growth, differentiation, gene expression, cells migration and the immune reactions. We examined the impact of RvD1 injection on the extracellular secretome, 1 day post force application (**Figures 2A, B**), using the Proteome Profiler assay which detects 111 extracellular signaling molecules. We found differences, mostly in the expression of cytokines and chemokines. RvD1 reduced the expression of pro-inflammatory cytokines such as-IL-6, IL-7, etc. and increased the expression of anti-inflammatory cytokines such as IL-4, IL-10, IL-13, IL-1ra, etc., indicating a shift to an anti-inflammatory phenotype. Interestingly, IL-15 cytokine (a regulatory cytokine for T and NK cell activation) showed a dramatic increase in response to RvD1 treatment (**Figure 2C**).

The chemokine profile showed increased expression of chemokines associated with recruitment of macrophages and resolution of inflammation (such as CCL6, 17, 19, 22, etc.).

Overall, these proteome changes indicate that RvD1 promotes resolution of OTM-induced sterile inflammation, similarly to its effects in pathogen-related inflammatory processes.

Since the Proteome Profiler is semi-quantative assay and doesn’t allow statistical analysis we also included a validation of selective proteins using ELISA assays: IL-1ra (29), and CCL6 (30). These cytokines were selected due to the abundant information on their function and their specific functions in inflammation.

ELISA results corroborated with the profiler assay, with higher levels of IL-1ra and CCL6 in the RvD1 treated group compared with saline controls (3570 ± 185.7 pg/ml vs. 2581 ± 455.9, respectively; p<0.05 and 114.7 ± 9.112 vs. 79.33 ± 14.89, respectively; p<0.05 (**Figures 2G, H**). IL-6 levels were lower than the ELISA detection threshold, therefore these results were excluded.

### RvD1 affects immune cell migration in the acute OTM phase

**Figure 3:**
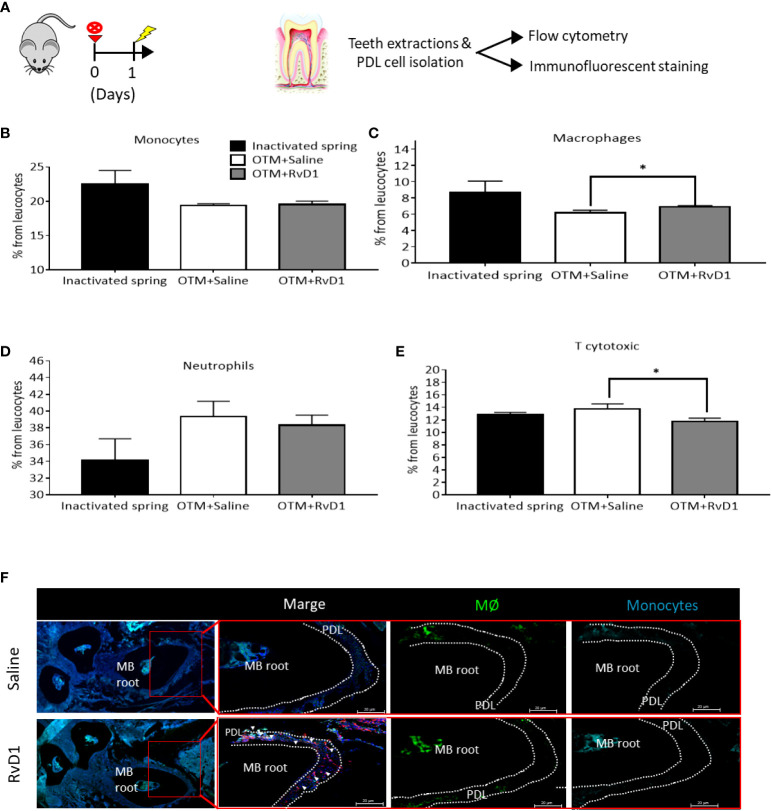
RvD1 Affects Immune Cell Migration in The Acute Phase of OTM **(A)** Schematic time course of OTM+ RvD1 treated mice (n=6), OTM+ Saline treated mice (n=6) and inactivated spring, after 1 day. **(B-E)** Immunotyping by FACS analysis of PDL following OTM + single injection of RvD1 vs. Saline treatment and inactivated spring, after 1 day. Graphs display the results for: **(B)** monocytes (CD45^+^,CD64^+^); **(C)** macrophages (CD45^+^,F4/80^+^); **(D)** neutrophils (CD45^+^,Ly6g^+^); and **(E)** T cytotoxic cells (CD45^+^,CD3^+^, CD8^+^) expression after OTM + RvD1 (dark gray bars) or Saline treatment (white bars), compared with the control baseline of inactivated springs (black bars), *p<0.05. **(F)** IF staining of macrophages and monocytes recruited to the PDL tissue in response to OTM + RvD1 compared to the Saline treatment control.

We further analyzed the RvD1 effect on immune cells recruitment, 1 day after force application (**Figure 3A**). FACS analysis of PDL cells (22) showed that RvD1 induced a mild but statistically significant increase in the percentage of macrophages and a reduction in the percentage of T cytotoxic cells out of the whole population of recruited PDL leukocytes, in comparison with saline injection controls (macrophages: 6.9% ± 0.16 vs 6.19% ± 0.31; T cytotoxic cells: 11.78% ± 0.49 vs 13.76% ± 0.81, respectively, p<0.05; **Figures 3C, E**). Monocytes and neutrophils did not show differences between the groups (**Figures 3B, D**). Immunofluorescence staining confirmed elevated numbers of macrophages and an insignificant change in monocytes (**Figure 3F**).

### RvD1 directly reduces osteoclastogenesis and RANK expression in prolonged OTM

**Figure 4:**
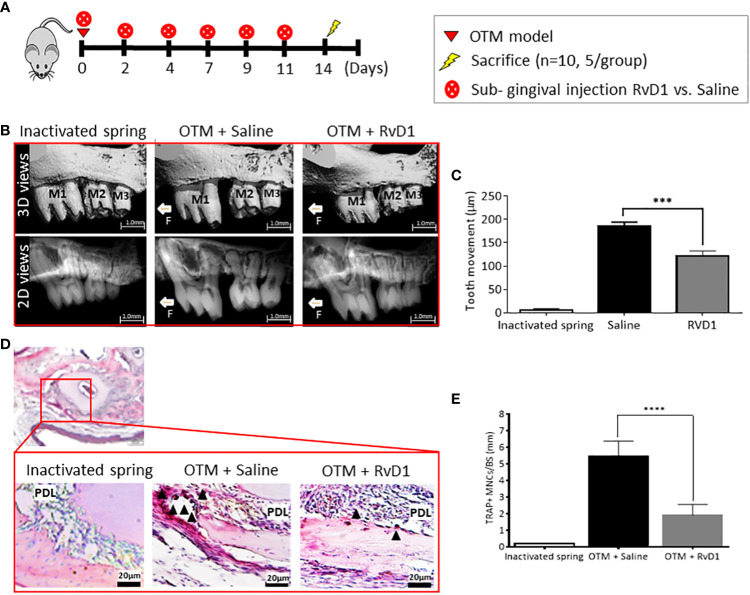
RvD1 Directly Reduces Osteoclastogenesis in Prolonged OTM **(A)** Schematic time course of OTM + RvD1 (n=6) vs. OTM + Saline (n=6) subgingival injections, every other day for 14 days. **(B)** OTM distance measurements with serial subgingival injections of RvD1 vs. Saline, 14 days after force application. 3D images taken by µCT (top); 2D images taken by µCT (bottom). **(C)** OTM distance analysis (***p<0.001). **(D)** TRAP staining for maxillae sections, 14 days post force application. TRAP positive multinucleated cells (arrows), as observed under the photomicroscope magnification. **(E)** Numerical analysis for TRAP positive multinucleated cells in OTM+RvD1 group compared with controls (****p< 0.0001).

We administrated 6 sub-gingival serial injections (**Figure 4A**) of RvD1 or saline, during 14 days of OTM. 2D and 3D measurements (**Figures 4B, C**) showed a statistically significant deceleration of OTM in RvD1 treated mice compared with the saline control group (121.0 ± 11.6 μm vs. 185.8 ± 8.7 μm, respectively; p<0.001). We proved that the local administration of RvD1 significantly reduced the number of osteoclasts (1.9 ± 0.3 cells vs. 5.4 ± 0.3 cells, P<0.0001; **Figures 4D, E**).

### RvD1 directly reduces osteoclastogenesis *via* RANK downregulation, without cytotoxic effects

Proteome profiler and FACS results provided a hint to the possible role of RvD1 not only in inflammation but also in BR. Therefore, we further aimed to investigate whether RvD1 has a direct effect on osteoclast cells.

Osteoblasts and stromal stem cells express RANK ligand (RANKL), which binds to its receptor RANK, expressed at very high levels on osteoclast precursors. RANKL-RANK interaction regulates the differentiation of precursors into multinucleated osteoclasts, osteoclasts activation and survival (31).

**Figure 5:**
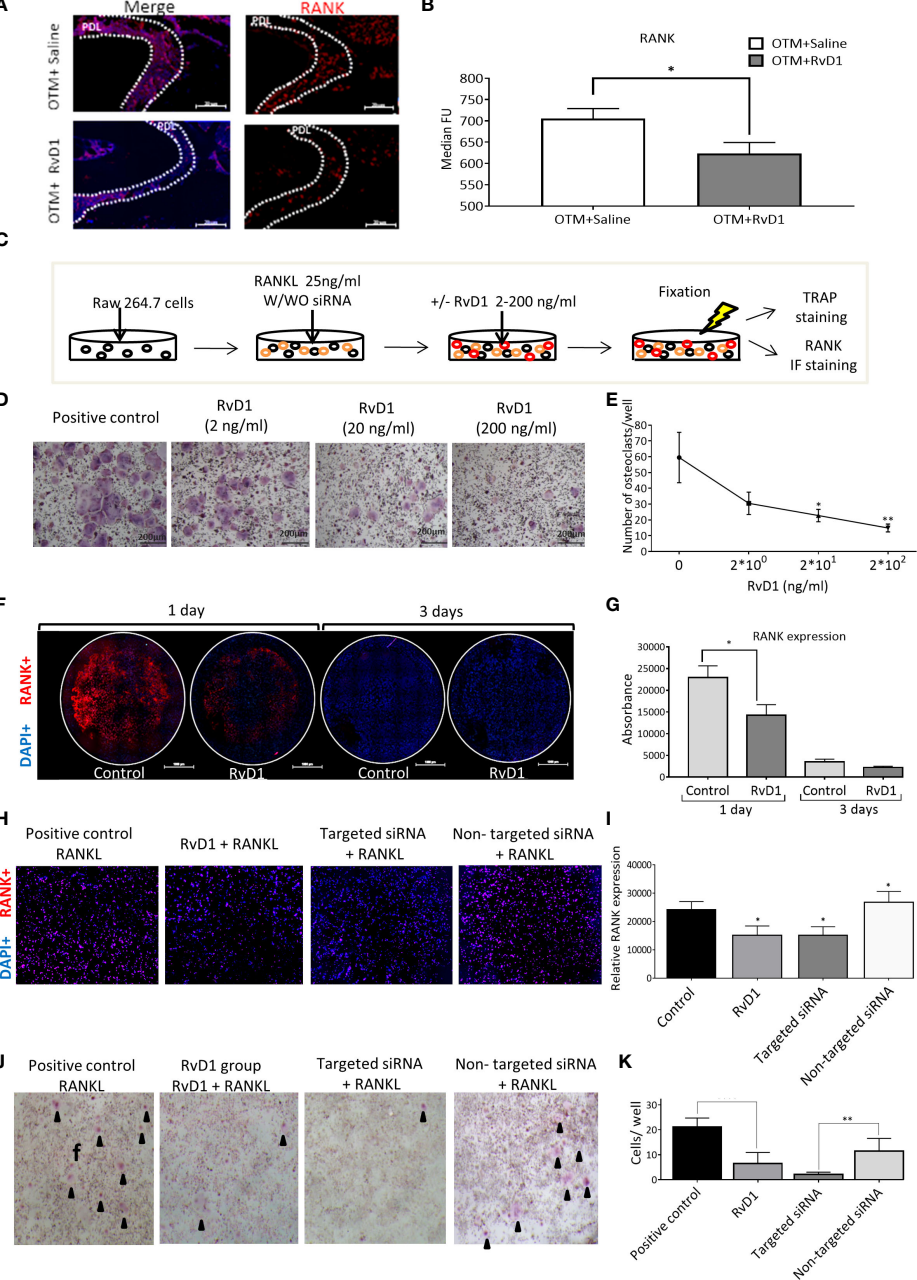
RvD1 Directly Reduces Osteoclastogenesis *via* RANK Downregulation, without Cytotoxic Effects **(A)** Immunotyping by FACS analysis of PDL following OTM + single RvD1 vs. Saline treatment and inactivated spring, after 1 day. Graphs display RANK expression after OTM + RvD1 (dark gray bars) or Saline treatment (white bars), compared with the control inactivated springs, after 1 day (black bars); *p<0.05. **(B)** IF staining demonstrates RANK expression in PDL after RvD1 compared to the Saline control treatment. **(C)** Schematic time course of RAW 264.7 cells differentiation to osteoclasts cells with/without RvD1 stimulation and siRNA transfection, after 3 days. **(D)** The *in vitro* effect of RvD1 on osteoclastogenesis. Digital images of TRAP staining of RAW 264.7 cells treated with RvD1 gradient compared with positive control (X20 magnification). **(E)** Numerical analysis for TRAP positive multinucleated cells in RvD1 groups compared with the positive control (0 ng/ml). *p<0.05; **p<0.01. **(F)** RANK expression in RAW 264.7 cells following RvD1 treatment vs. control, after 1 day of incubation. Digital images of RANK (in red) and DAPI (in blue) fluorescence staining (X10 magnification). **(G)** Fluorescence absorbance of PE anti-mouse CD265 antibody indicating RANK expression. **(H)** RANK expression in RAW 264.7 cells 24 hours following anti RANK siRNA transfection vs. RvD1 treatment and control. Digital images of RANK (in red) and DAPI (in blue) fluorescence staining (X20 magnification). **(I)** Fluorescence absorbance of PE anti-mouse CD265 antibody indicating RANK expression. *p<0.05. **(J)** The *in vitro* effect of RvD1 and siRNA transfection on osteoclastogenesis. Digital images of TRAP staining of RAW 264.7 cells treated with RvD1 compared with positive control and siRNA transfected cells (X4 magnification). **(K)** Numerical analysis for TRAP positive multinucleated cells. **p<0.01.

We examined the RANK expression following 1 day of OTM and a single injection of RvD1 or saline, compared with the endogenous expression in the inactivated spring control group. Since RANK is a type 1 transmembrane protein, we were able to stain and identify the expression of the receptor in response to the various treatments. RvD1 significantly reduced RANK expression, compared with saline control (623.4 ± 25.7 MFU vs 705.1 ± 23.7 MFU, respectively, p<0.05; **Figures 5A, B**).

Next, we aimed to investigate the RvD1 mechanism of action on osteoclasts, *in vitro*. The model was based on the formation of osteoclasts following murine RAW 264.7 cell line exposure to RANKL (**Figure 5C**). Administration of various quantities of RvD1 induced a dose-dependent reduction of TRAP positive multinucleated cells (59.5 ± 15.9 cells in the control group; 30.5 ± 7.1 cells for 2 ng/ml RvD1; 22.7 ± 3.9 cells for 20 ng/ml RvD1, p<0.05; 14.9 ± 2.5 cells for 200 ng/ml of RvD1, p<0.01; **Figures 5D, E**). Based on these results, further experiments were performed using a dose of 200ng/ml RvD1. To verify that this reduction in osteoclastogenesis did not stem from a cytotoxic effect of RvD1, we performed a XTT based assay. Results showed that RvD1 had no cytotoxic effect on RAW 264.7 cells (**Appendix Figure 2**).

Consequently, we assumed that RvD1 has direct effect on osteoclasts cell *via* RANK receptor. To address this assumption, we investigated RANK expression on RAW cells, 1 day after exposure to RANKL and RvD1, as detailed in **Figure 5A**. Results show that RvD1 significantly reduced RANK expression on differentiating RAW cells after 1 day of incubation, compared with the controls (florescent intensity of 14283 ± 2395 vs. 22926 ± 2703, respectively; p<0.05; **Figures 5F, G**). No statistically significant differences were found after 3 days of incubation.

To provide evidence that RANK reduction is the only path by which RvD1 affects osteoclastogenesis, we included a siRNA transfection specific assay that specifically reduces RANK (*via *degrading mRNA after transcription and preventing its translation) without affecting any other processes, and compared its effect with that of RvD1.

The results showed that RANK targeted siRNA caused a significant reduction of RANK expression while the non-targeted siRNA had no effect, confirming its use as a control for the siRNA transfection. Importantly, RvD1 mimicked RANK targeted siRNA mechanism, by knocking down and significantly suppressing RANK expression on RAW 264.7 cells’ surface after 1 day, in comparison to the no RvD1 control group and to the non-targeted siRNA transfected cells (relative RANK expression of 15166 ± 3297 vs. 24275 ± 2817 and 26855 ± 3792, respectively; p<0.05). No significant differences were found between the control and the non-targeting siRNA groups (**Figures 5H, I**).

To corroborate the above results, we then compared the effect of RvD1 and siRNA transfection on osteoclastogenesis. RvD1 strongly inhibited osteoclasts differentiation similarly to the RANK targeted siRNA group (6.5 ± 1.8 cells and 2.2 ± 0.4 cells, respectively, versus 21.2 ± 1.4 cells for the positive control group, *p*<0.0001) (**Figures 5J, K**).

## Discussion

Previous studies revealed the pro-resolving and anti-inflammatory effects of resolvins in general and RvD1, in particular, in pathogen-induced inflammation (12). Remarkably, their role in sterile inflammation and BR has been scarcely studied (32, 33). In the current study, an OTM model, in which inflammation and BR are triggered by a mechanical force and not by an infective agent, was used to reveal the RvD1 mechanism of action in both acute (1 day post force initiation) and prolonged (14 days) phases of sterile inflammation and its effect on osseous tissue (2, 21).

We initially wondered whether RvD1 participates in OTM. Our results demonstrate for the first time that endogenous RvD1 is released locally in the acute phase of OTM showing a peak at 6 hours and a decrease to baseline levels after 24 hours post force application. Furthermore, a single injection of exogeneous RvD1 3 days post force application significantly reduced OTM, indicating that RvD1 plays an active role in cooling off the inflammatory process and the related bone resorption.

To understand the molecular mechanisms underlying the clinical effect of RvD1 in the acute phase, we conducted a site secretome profiling characterization, 1 day after force initiation. Local administration of RvD1 reduced the pro-inflammatory and increased the anti-inflammatory cytokines’ secretion, supporting its role in resolution of the acute inflammation. Interestingly, the cytokines which showed most changes play a critical dual role in promotion of innate and adaptive immunity *via* immune cell recruitment and activation (34) and in slowing down tissue remodeling *via* inhibition of metalloproteinases and cathepsins (35).

Noticeable increase occurred in the expression of several chemokines, such as CCL12/MCP-5 which has been shown to encourage inflammatory cell trafficking (36) and CCL17/TARC, which attracts primed CD4^+^ T cells (37). CCL19 is a critical regulator of T cell activation, induces a potent proinflammatory differentiation program in licensed dendritic cells (38), Importantly, it also stimulates (via CCR7) migration of bone marrow mesenchymal stem cells that can differentiate into osteoblasts (39). CCL22/MDC plays an important role in recruitment of Th2 cells and regulation of Th2 anti-inflammatory related immune responses (40).

An intriguing effect of RvD1 administration was the dramatic increase in IL-15, an innate proinflammatory cytokine mostly produced by macrophages and dendritic cells (41). A possible explanation might be that this cytokine has both pro and anti-inflammatory activities. Indeed, the anti-inflammatory potential of IL-15 has previously been described, suggesting a protective role against an exaggerated Th1 immune response in certain inflammatory conditions (42). The elevated levels of IL-15 are in accordance with the decrease in Th1 (IFN-γ) and the increase in Th2 cytokines (IL-4, IL-10) and CCL22 chemokine (40), as demonstrated in **Figure 2**.

In the context of bone biology, the secretome profiler showed a decrease in CCL21, which has been shown to promote osteoclast migration and resorption activity (43) and the *in vitro* migration and maturation of dendritic cells, which share their precursors with osteoclasts (44, 45). Furthermore, RvD1 significantly increased IL1-raIL1-ra has been shown to inhibit osteoclast formation (46). Furthermore, IL-1a and IL-6, which induce RANKL and osteoclasts marker expression (47, 48), were decreased.

Our results support Benabdoun et al. who demonstrated a decrease in bone and cartilage turnover markers in response to RvD1, in an arthritic sterile inflammation mouse model (19).

Altogether, these findings support a role for RvD1 in the interplay between sterile inflammation, the immune system, and the induced BR.

To examine the RvD1 role at the cellular level, we performed immune cells immunotyping in PDL (**Figure 3**). After 24h of OTM, we found a modest but significant upregulation in the number of recruited macrophages (F4/80^+^), which correlates with the increase in CCL12/monocyte chemotactic protein-5 (MCP-5), found in the secretome profiling. Our results on the effect of RvD1 on macrophages, in conjunction with data from previous studies (49, 50), confirm an indirect role of RvD1 in promoting clearance of the inflammation components.

An additional interesting finding was the RvD1-induced downregulation of T cytotoxic (CD3^+^; CD8^+^) migration, which strengthens previous data on its role in the control of adaptive immunity. Previous studies demonstrated that exogenous RvD1 regulates T-cell activation in choroid and retina (51) and that RvD1 reduce CD8^+^ and CD4^+^ cell activation as well as prevent Th1 and Th17 cell differentiation from naïve T cells (52).

In the present study, we found no significant differences in monocyte and neutrophils, in contrast to previous studies in pathogen-induced peritonitis models which showed that RvD1 reduces neutrophil infiltration (53). These contrasting results may stem from the reduced intensity of OTM-triggered sterile versus pathogen-related inflammation.

Next, we aimed to evaluate the effect of RvD1 in prolonged OTM inflammation, by administrating serial doses over 14 days. The results showed a continuous reduction in OTM, attributable to a reduction in the number of TRAP^+^ cells.

The significant changes in the expression of factors associated with BR suggested a direct effect of RvD1 on osteoclasts. Our FACS results demonstrated that RvD1 significantly decreased RANK expression after 1 day of OTM, supporting its direct effect on pre- and mature osteoclasts.

To further understand the mechanism of action of RvD1 on osteoclasts and confirm that RANK reduction is the prominent path by which RvD1 affects osteoclastogenesis, we established an *in-vitro* model which included an anti-RANK siRNA transfection specific assay. RvD1 reduced osteoclast differentiation in a dose dependent manner, without cytotoxic effect. The effect of RvD1 was achieved by direct suppression of RANK expression on the cells surface, mimicking the RANK targeted siRNA mechanism.

Our findings are consistent with Yuan et al. who reported on a dose dependent inhibitory effect of RvD1 on osteoclastogenesis in a sRANKL-induced differentiation of bone marrow-derived macrophages (BMMs) into osteoclasts model, *in vitro*. Noteworthy to mention, 10 µM RvD1 added to the cell culture reduced osteoclastogenesis by 75%, similarly to our results (54).

The few studies which investigated the role of resolvins in bone biology mainly focused on RvE1. RvE1 promoted bone preservation under local inflammatory conditions (55) and modulated osteoclast differentiation and BR by direct actions on bone, rescuing OPG production and restoring a favorable receptor activator of RANKL/OPG ratio (15).

Lately, the interest in RvD1 increased not only due to its ability to neutralize inflammatory and catabolic tissue insults, but also due to evolving data showing its ability to repair injured tissues and most importantly to promote their regeneration (18).

**Figure 6:**
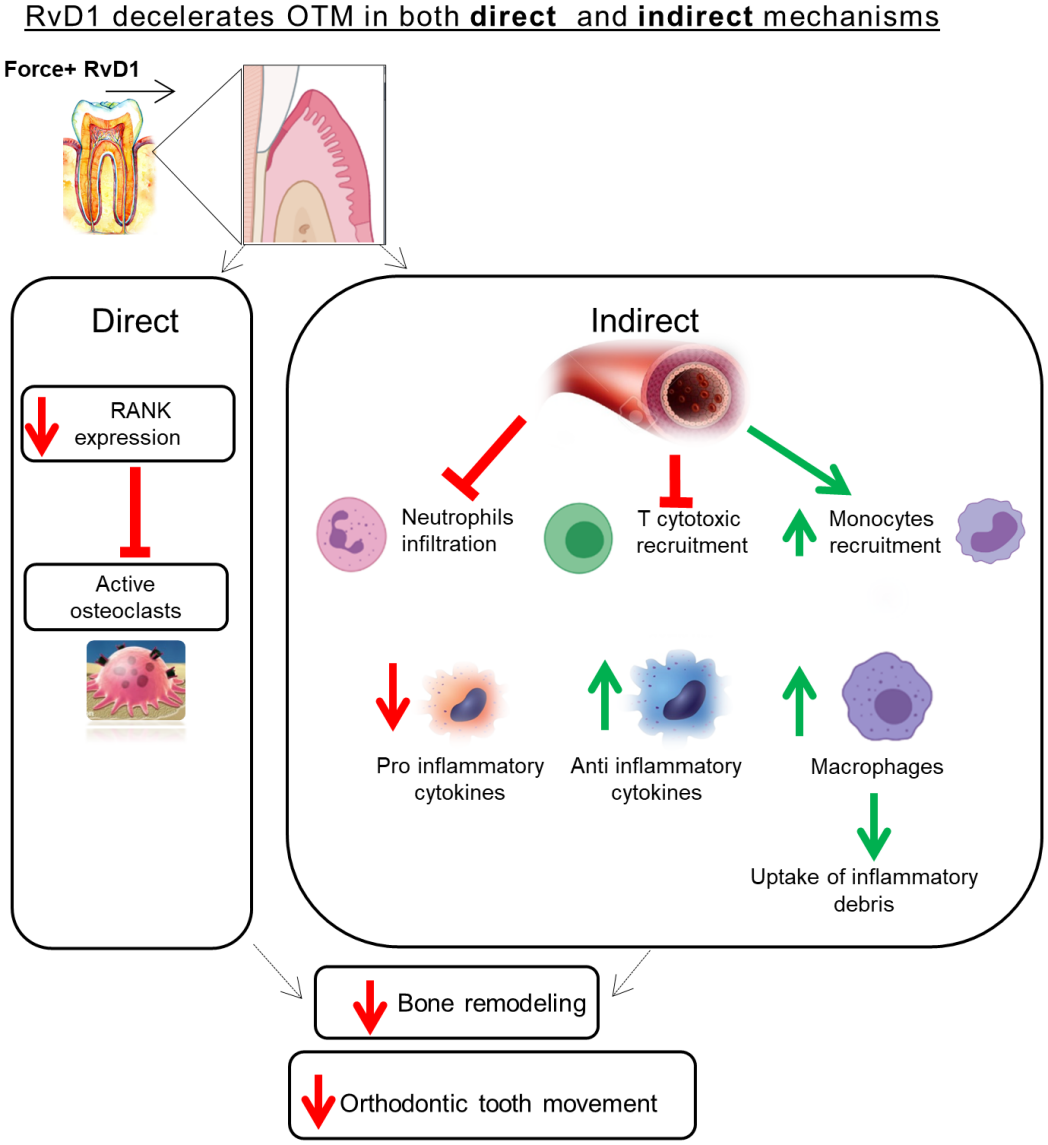
Schematic illustration of the RvD1 mechanism of action in OTM.

The current study specifically focused on RvD1 mechanism of action in OTM induced sterile inflammation and BR. It provides evidence that RvD1 has a dual role: in the acute inflammation phase, RvD1 has an indirect pro-resolution and anti-inflammatory effect through recruitment of inflammatory cells and mediators; in the prolonged inflammation phase RvD1 suppresses osteoclastogenesis *via* direct downregulation of RANK expression. The RvD1 mechanism of action in OTM is schematically illustrated in **Figure 6**. Future studies are still needed to investigate to effect of RvD1 on Osteoblast cells.

The limitations of this study include the use of male mice only (to eliminate the possible hormonal and sex cycle effects on the bone metabolism occur in female mice), the unavailability of Mass spec assay for proteomics, and the moderate sample size due to the ethics committee limitations. Gender differences should be further investigated in the future and finally, the above results should be validated in humans.

Our data supports RvD1 as a promising bioagent to control OTM associated inflammation and prevent pathologic bone destruction, due to its ‘immunoresolvent’ as well as ‘osteoresolvent’ effects in osteo-inflammatory resolution.

## Data availability statement

The original contributions presented in the study are included in the article/**Supplementary Material**. Further inquiries can be directed to the corresponding author.

## Ethics statement

The animal study was reviewed and approved by IACUC of the Hebrew University (MD-18-15426-4).

## Author contributions

YK, OL-T, DP and SC contributed to conception of the manuscript, study design, data analysis and interpretation and drafted the manuscript. YK and OL-T contributed to data acquisition, analysis, and interpretation. JG and YB contributed to conception of the manuscript and drafted the manuscript. YK, OL-T, JG, SW, YM, AM, AL contributed to data acquisition. SC takes responsibility for the integrity of the data analysis. All authors gave their final approval and agreed to be accountable for all aspects of the work.

## Funding

This work was supported by the Israel Science Foundation and Dr. Izador I. Cabakoff Research Endowment Fund foundation.

## Conflict of interest

The authors declare that the research was conducted in the absence of any commercial or financial relationships that could be construed as a potential conflict of interest.

## Publisher’s note

All claims expressed in this article are solely those of the authors and do not necessarily represent those of their affiliated organizations, or those of the publisher, the editors and the reviewers. Any product that may be evaluated in this article, or claim that may be made by its manufacturer, is not guaranteed or endorsed by the publisher.
