# Supplementary figures and images for: Resolvin D1 shows osseous-protection via RANK reduction on monocytes during orthodontic tooth movement
Source: Front Immunol. 2022 Oct 7;13:928132. doi: 10.3389/fimmu.2022.928132 (PMC9585452; doi:10.3389/fimmu.2022.928132)

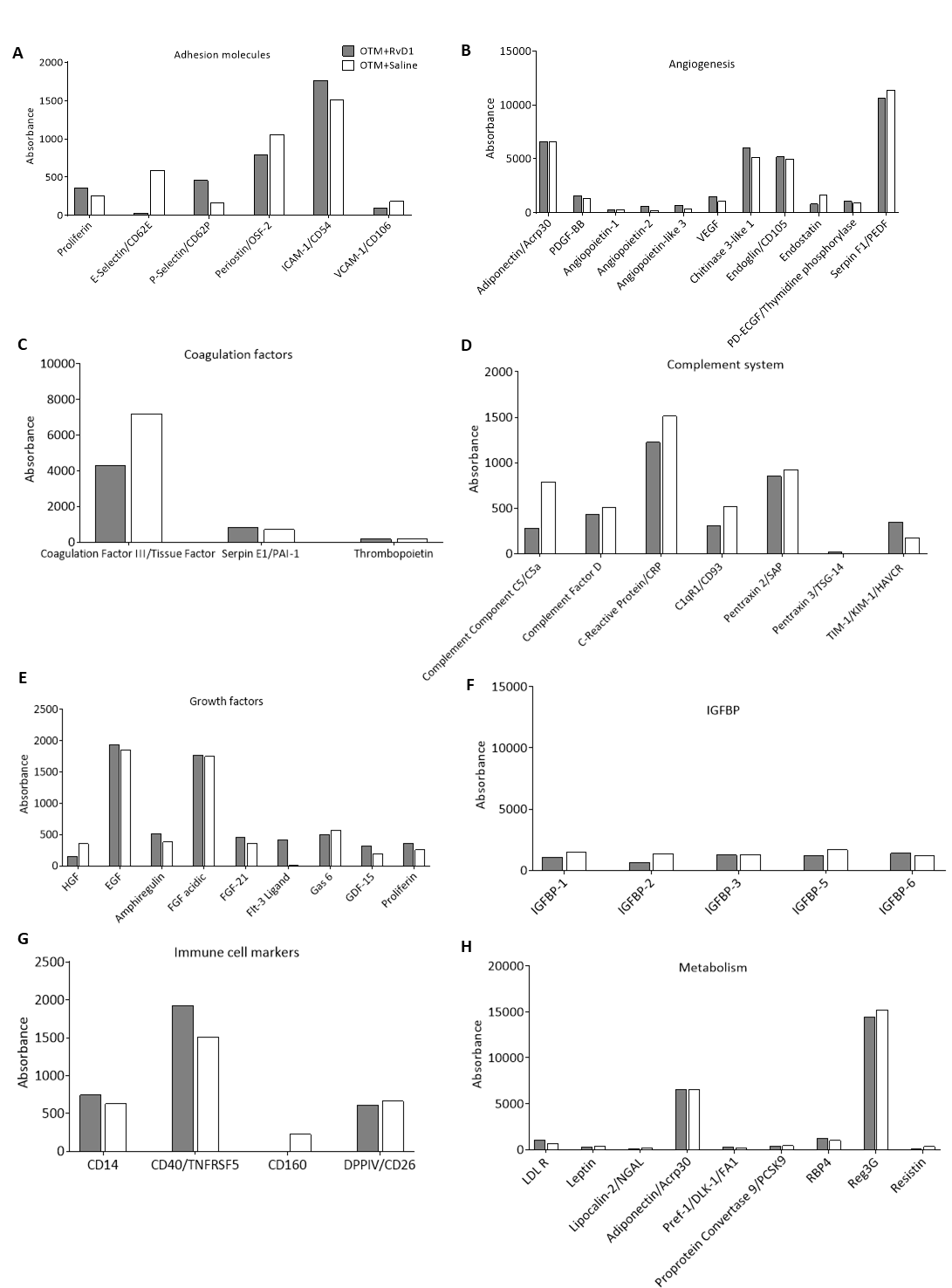

Supplement: Supplementary file 1 [file Image_1.tif]

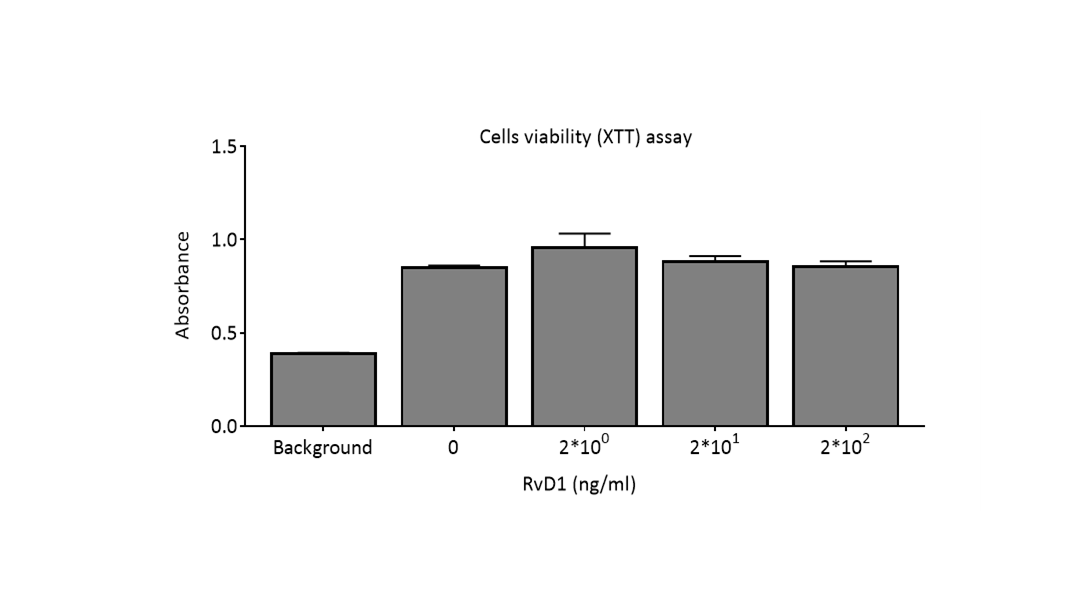

Supplement: Supplementary file 2 [file Image_2.tif]

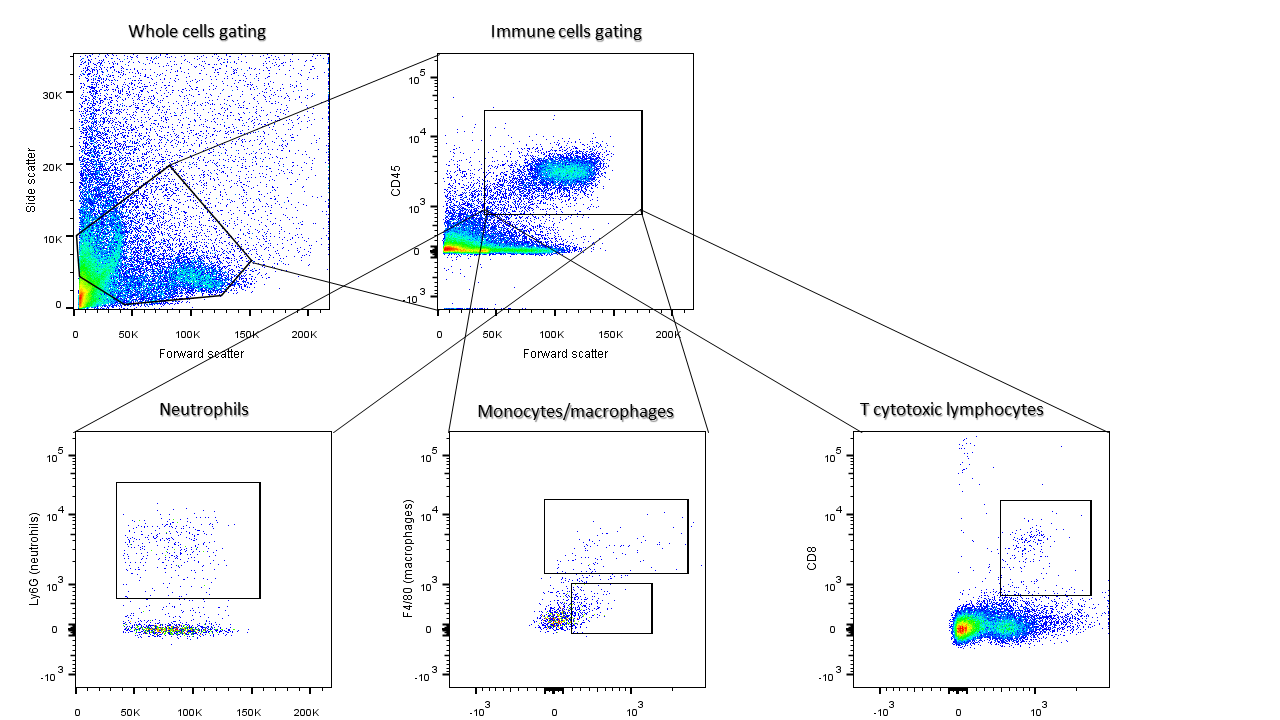

Supplement: Supplementary file 3 [file Image_3.tif]
